# Surveillance for early stages of colon cancer: potentials for optimizing follow-up protocols

**DOI:** 10.1186/s12957-015-0674-7

**Published:** 2015-08-28

**Authors:** Elisa Gilardoni, Davide Paolo Bernasconi, Silvia Poli, Mattia Garancini, Margherita Luperto, Nicola Zucchini, Giorgio Bovo, Mauro Totis, Alvaro Bugatti, Luca Gianotti

**Affiliations:** Department of Surgery and Translational Medicine, Milano-Bicocca University, San Gerardo Hospital, Monza, Italy; Department of Health Science, Centre of Biostatistics for Clinical Epidemiology, Milano-Bicocca University, Monza, Italy; Unit of Pathology, San Gerardo Hospital, Monza, Italy; Department of Surgery, San Gerardo Hospital (4° piano A), Via Pergolesi 33, 20052 Monza, Italy

**Keywords:** Colon cancer, Surveillance, Follow-up, Early stage, Surgery

## Abstract

**Background:**

Although several meta-analyses showed the positive effects of follow-up on the prognosis of colon cancer (CC), international guidelines are not in accordance on appropriate tests and their time frequency to optimize surveillance. Furthermore, stratified strategies based upon risk grading have not been implemented. This approach may be useful to rationalize resources.

**Methods:**

From 2006, all patients operated for an early stage CC (I, IIA, IIB) according to the 7th edition of the AJCC-2010 classification entered in a prospective surveillance program in accordance to our local guidelines. Patients who underwent surgical resection after 2009 have been excluded to guarantee at least a 5-year follow-up. Classic histopathologic prognostic factors such as grade, T and N status, lymphatic and vascular invasion were assessed. Moreover, tumor budding and tumor-to-stroma proportion were evaluated.

**Results:**

We had complete records of 196 patients. Distribution was as follows: 65 (33.2 %) in stage I, 122 (62.2 %) in stage IIA, and 9 (4.6 %) in stage IIB. Eleven patients (5.6 %) had a disease recurrence (local or distant). The median recurrence time was 20 months (range 6–48). Nine patients (82 %) had recurrence with 24 months, and 91 % were asymptomatic and detected by ultrasound or CT scan. According to the log-rank test, the risk factors with significant effect on the disease-free survival (DFS) were the number of lymph nodes <12 (*p* = 0.027) and the vascular invasion (*p* = 0.021), while for the overall (OS), only the vascular invasion was significant (*p* = 0.043). By the univariate and multivariate analyses, DSF was significantly lower in patients with less than 12 nodes removed, with vascular invasion, and with left of double cancer. OS was negatively affected only by vascular invasion despite the hazard ratios were similar to DSF. Stage IIB was associated with a threefold-increased risk of reduced OS and DSF.

**Conclusions:**

Stages I and IIA appear to behave similarly and should be considered as true early stages. The detection of fibrosis and budding do not seem to add valuable information for prognosis. In early CC stages, the surveillance program should be maximized within the first two years.

## Background

The prognosis of colon cancer (CC) has improved over the years due to the earlier detection of the disease and improved surgical techniques and more effective chemotherapy.

In the management of early stages of CC, surgery alone remains the best treatment option. Despite the excellent prognosis of early stages, the possibility of local recurrence and appearance of metachronous metastases exists, and it is directly correlated with some well known risk factors such as occlusion or perforation at presentation, TNM classification, vascular and lymphatic invasion, number of nodes retrieved, tumor grading, KRAS, BRAF mutation, and microsatellite instability [1–5]. Recently, other histopathologic features of CC such as tumor budding [6–8] and the presence of fibrosis [9, 10] have been recognized as important negative predictive factors. However, the prognostic performance of these new elements has not been fully validated in all cancer stages.

Surveillance remains a cornerstone approach to detect recurrence at an early stage [11, 12] and consequently to plan further therapeutic strategies. Although several meta-analyses have been performed on the positive effects of the follow-up on CC prognosis [13, 14], international guidelines are not in accordance on appropriate tests and their time frequency to optimize surveillance [15–18]. Furthermore, stratified strategies based upon risk scaling have not been implemented. This approach may be useful to rationalize resources.

The aims of this study were to identify predictors of recurrent disease and long-term survival among subjects operated for early stages (I, IIA, IIB) of CC defined according to the latest edition of the American Joint Committee on Cancer (AJCC) [19] and to determine potential recommendations for a optimize follow-up protocol.

## Methods

Since 2006, we prospectively recorded in an electronic database all the clinical and pathological data, and the office visits of all patients operated for CC at the Department of Surgery of the Milano-Bicocca University-San Gerardo Hospital.

All patients entered in a surveillance program in accordance to our local guidelines (Table 1). All patients underwent contrast-enhanced abdominal ultrasound. In cases of unclear results of CEUS imaging, a contrast-enhanced CT scan was done.

**Table 1:** Routine surveillance protocol according the local guidelines

Months after operation	Medical history and physical examination	Complete blood count (CEA, CA 19–9 included)	Contrast-enhanced abdominal ultrasound	Abdominal and chest computed tomoghapy	Colonoscopy
6	X	X	X		
12	X	X		X	X
18	X	X	X		
24	X	X		X	
30	X	X	X		
36	X	X		X	X
48	X	X	X		
60	X	X			X

From this database, we retrospectively selected patients with a potentially curative resection and an early stage disease (I, IIA, IIB) according to the 7th edition of the AJCC-2010 classification [19] and operated between 2006 and 2009. We defined a radical resection (R0) when the resection margins were ≥5 cm, and circumferential margins were not involved. Patients who underwent surgical resection after 2009 have been excluded to guarantee at least a 5-year follow-up.

All cases have been reviewed by two GI pathologists (GB and NZ) at double-headed microscope to confirm the following histopathologic prognostic factors: grade, T and N status, lymphatic and vascular invasion. Tumor budding (isolated single cancer cells or cluster composed of fewer than five neoplastic cells) has been evaluated in each tumor as described by Ueno et al. [6, 7]; fields with most prominent budding at the tumor invasive margin were selected and a count of the foci of budding was made with a ×20 objective. Tumor budding was considered positive when bud count was higher than 9. Tumor-to-stroma proportion has been evaluated applying Huijbers et al. criteria [9]. The stroma percentage has been estimated at the most invasive area of each tumor at ×10. Each case has been given a tenfold scoring percentage and further grouped into the high-stroma (>50 %) or low-stroma (≤50 %) category for statistical analysis.

### Statistics

Descriptive statistics of the risk factors considered were computed separately for the patients with and without recurrent disease. Two end-points were considered: disease-free survival (DFS), defined as the time elapsed from the intervention to relapse or death in absence of relapse or end of the follow-up, and overall survival (OS), defined as the time elapsed from the intervention to death by any cause or end of the follow-up. The Kaplan-Meier survival curves were estimated for the two end-points and for each risk factor, and were compared using the log-rank test. Finally, the effect of the risk factors on the two end-points was evaluated with both univariate and multivariate regression analyses, using the Cox model. All *p* values <0.05 were considered significant. The R software version 3.0.2 was used for statistical analyses.

## Results

In the study period, a total number of 567 patients were operated on for CC. Three hundred and thirty-eight patients were exclude, 37 in stage 0; 48 in stage IIC; 144, in stage III; 105 in stage IV. Two hundred and thirty-three subjects with stage I, IIA, or IIB were selected. Thirty-four patients (14.6 %) were excluded for incomplete or lost at follow-up and three patients for incomplete data for pathology examination. A final population of 196 patients with complete records was distributed as follows: 65 (33.2 %) in stage I, 122 (62.2 %) in stage IIA, and 9 (4.6 %) in stage IIB.

Eleven patients (5.6 %) had a disease recurrence (local or distant). The characteristics of the overall population and of the patients with and without recurrence are described in detail in Table 2. No significant differences were observed when patients with and without recurrence were compared with the exception of cancer site. Subjects with tumor of the left colon and multiple cancer location were at significant higher risk of recurrence. We observed a trend toward a significant increase of risk for patients with less than 12 nodes retrieved, presence of vascular and lymphatic invasion and high presence of fibrosis.

**Table 2:** Characteristics of the overall population and of patients with or without recurrence

Characteristic	Total	Recurrence	Non recurrence	*p* value
Overall patients	196	11 (5.6)	185 (94.4)	
Gender				
Male	117 (59.7)	5 (45.5)	112 (60.5)	0.356
Female	79 (40.3)	6 (54.5)	73 (39.5)	
Median age (range), years	70 (40–89)	71 (63–81)	70 (40–89)	0.262
ASA score				
1	7 (3.6)	0	7 (3.8)	0.661
2	116 (59.2)	7 (63.6)	109 (58.9)	
3	66 (33.7)	3 (27.3)	63 (34.1)	
4	7 (3.6)	1 (9.1)	6 (3.2)	
Tumor site				
Right colon	83 (42.3)	2 (18.2)	81 (43.8)	0.009
Trasversum colon	17 (8.7)	0	17 (9.2)	
Left colon	94 (48.0)	8 (72.7)	86 (46.5)	
>1 location	2 (1.0)	1 (9.1)	1 (0.5)	
T				
1	10 (5.1)	0	10 (5.4)	0.772
2	56 (28.6)	3 (27.3)	53 (28.6)	
3	121 (61.7)	7 (63.6)	114 (61.6)	
4a	9 (4.6)	1 (9.1)	8 (4.3)	
Stage				
1	65 (33.2)	3 (27.3)	62 (33.5)	0.726
2A	122 (62.2)	7 (63.6)	115 (62.2)	
2B	9 (4.6)	1 (9.1)	8 (4.3)	
Grading				
1	15 (7.7)	0	15 (8.1)	0.349
2	166 (84.7)	11 (100)	155 (83.8)	
3	15 (7.7)	0	15 (8.1)	
Lymph nodes retrieved				
> = 12	153 (78.1)	6 (54.5)	147 (79.5)	0.066
<12	43 (21.9)	5 (45.5)	38 (20.5)	
Vascular invasion				
yes	9 (4.6)	2 (18.2)	7 (3.8)	0.083
no	187 (95.4)	9 (81.8)	178 (96.2)	
Lymphatic invasion				
yes	32 (16.3)	4 (36.4)	28 (15.1)	0.084
no	164 (83.7)	7 (63.6)	157 (84.9)	
Growth				
Infiltrative	108 (55.1)	7 (63.6)	101 (54.6)	0.784
Expansive	88 (44.9)	4 (36.4)	84 (45.4)	
Lymphocyte infiltration				
no	60 (30.6)	6 (54.5)	54 (29.2)	0.301
small	19 (9.7)	1 (9.1)	18 (9.7)	
small-moderate	71 (36.2)	2 (18.2)	69 (37.3)	
moderate	22 (11.2)	0	22 (11.9)	
severe	24 (12.2)	2 (18.2)	22 (11.9)	
Setting				
elective surgery	180 (91.8)	11 (100)	169 (91.4)	0.605
emergency	16 (8.2)	0	16 (8.6)	
Blood transfusions				
no	164 (83.7)	10 (90.9)	151 (81.6)	0.707
yes	35 (16.3)	1 (9.1)	34 (18.4)	
Fibrosis				
10	100 (51.0)	3 (27.3)	97 (52.4)	0.054
20	28 (14.3)	2 (18.2)	26 (14.1)	
30	22 (11.2)	2 (18.2)	20 (10.8)	
40	12 (6.1)	1 (9.1)	11 (5.9)	
50	12 (6.1)	0	12 (6.5)	
60	8 (4.1)	0	8 (4.3)	
70	11 (5.6)	2 (18.2)	9 (4.9)	
80	3 (1.5)	1 (9.1)	2 (1.1)	
90	0	0	0	
100	0	0	0	
Fibrosis grade				
High (> = 50 %)	34 (17.3)	3 (27.3)	31 (16.8)	0.408
Low (<50 %)	162 (82.7)	8 (72.7)	154 (83.2)	
Budding				
High (> = 10 foci)	38 (19.4)	4 (36.4)	34 (18.4)	0.229
Low (<10 foci)	158 (80.6)	7 (63.6)	151 (81.6)	

Data are number of patients (%) or median (range) when specified

**Fig. 1:**
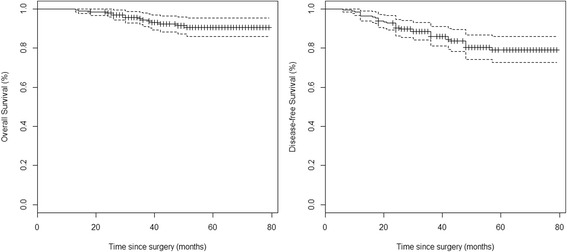
Kaplan-Meier curves for overall survival and disease-free survival. Marks at censoring times. Bands are 95 % confidential intervals

**Fig. 2:**
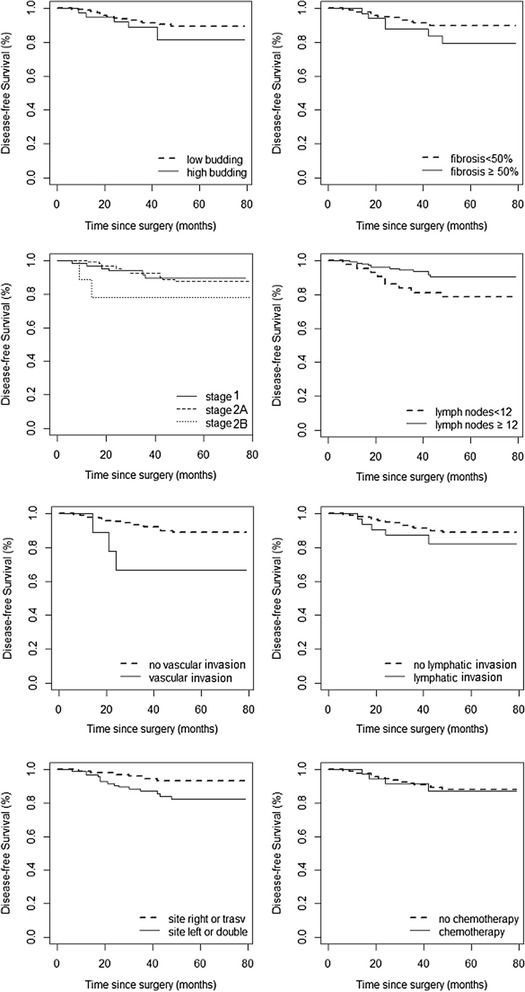
Kaplan-Meier curves for disease-free survival stratified for specific risk factors such as budding, fibrosis, staging, number of nodes, vascular and lymphatic invasion, tumor site, and adjuvant chemotherapy

**Fig. 3:**
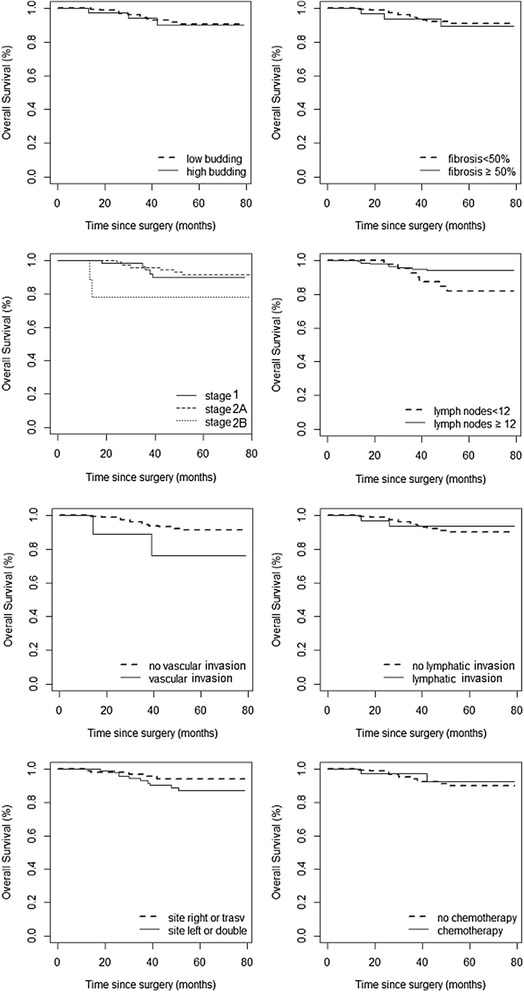
Kaplan-Meier curves for overall survival stratified for specific risk factors such as budding, fibrosis, staging, number of nodes, vascular and lymphatic invasion, tumor site and adjuvant chemotherapy

Table 3 shows the characteristics of the patients with recurrence, the site and timing of recurrence and the diagnostic tools used to prove it. Adjuvant chemotherapy was prescribed in 2 cases: one for cancer perforation and one for the small number of lymph nodes removed (*n* = 5) and massive lymphatic/vascular invasion. The mean recurrence time was 20 months from surgery (range of 6–48 months). In nine out of eleven patients (81.8 %), recurrence was within 24 months, and in all cases but one (90.9 %), recurrence was asymptomatic and detected by ultrasound or CT scan. Two patients had local recurrence, three pulmonary, one local and pulmonary, and five hepatic metastases. The Kaplan-Meier estimates (95 % CI) of the DFS and OS on the whole sample at 5 years after diagnosis were 0.878 (0.829–0.929) and 0.905 (0.860–0.953), respectively (Fig. 1). According to the log-rank test, the risk factors with a statistically significant effect on the DFS were the number of lymph nodes <12 (*p* = 0.027) and the vascular invasion (*p* = 0.021), while for the OS only the vascular invasion was significant (*p* = 0.043). These results are shown graphically by the Kaplan-Meier curves of Fig. 2 (DFS) and Fig. 3 (OS).

**Table 3:** Characteristics of the eleven patients with recurrent disease

Patient	Initial stage	Adjuvant chemotherapy after surgery	Site of recurrence	Presence of symptoms	Timing of recurrence (months)	Diagnostic tool
1	I	No	Liver	No	6	CEUS and CT
2	I	No	Local and lung	No	24	CEUS and CT
3	IIA	No	Lung	No	48	CT
4	IIA	No	Liver	No	18	CEUS and CT
5	IIA	Yes	Lung	No	18	CT
6	IIA + I	No	Liver	No	18	CT
7	IIA	No	Liver	Abdominal pain	42	CEUS and CT
8	IIA	Yes	Lung	No	24	CT
9	IIA	No	Local	No	12	CT
10	I	No	Local	No	12	Colonoscopy
11	IIB	No	Liver	No	12	CT

*CEUS* contrast-enhanced ultrasound

*CT* computed tomography

By the univariate analysis, DSF was significantly lower in patients with less than 12 nodes removed, with vascular invasion, and with left or double cancer. From a statistical point of view, OS was negatively affected only by vascular invasion despite the hazard ratios being similar to DSF. Stage IIB was associated with a threefold-increased risk of reduced OS and DSF, but the data might have generated bias for the small number of patients. By the multivariate analysis, the risk factors for decreased OS and DSF confirmed the results of the univariate analysis but only left and double cancer reached statistical significance (Table 4).

**Table 4:** Risk factors analyzed by univariate and multivariate (Cox regression models) for disease-free survival (DFS) and overall survival (OS)

	Univariate analysis		Multivariate analysis	
	DFS HR (95 % CI)	OS HR (95 % CI)	DFS HR (95 % CI)	OS HR (95 % CI)
Gender				
Female	1	1	1	1
Male	0.59 (0.25–1.40)	0.57 (0.21–1.57)	0.41 (0.16–1.05)	0.41 (0.14–1.25)
Age, years	1.06 (1.01–1.12)	1.09 (1.02–1.16)	1.07 (1.01–1.12)	1.09 (1.02–1.16)
Budding				
No	1	1	1	1
Yes	1.72 (0.67–4.44)	1.09 (0.31–3.85)	1.22 (0.35–4.16)	1.06 (0.23–4.87)
Fibrosis				
<50 %	1	1	1	1
> = 50 %	1.98 (0.77–5.10)	1.23 (0.35–4.35)	1.21 (0.37–3.94)	1.01 (0.22–4.61)
Stage				
1	1	1	1	1
2A	1.11 (0.42–2.93)	0.80 (0.26–2.46)	1.08 (0.35–3.30)	1.13 (0.31–4.13)
2B	2.89 (0.58–14.35)	3.68 (0.71–19.00)	4.07 (0.63–26.08)	5.81 (0.77–43.92)
Lymph nodes retrieved				
> = 12	1	1	1	1
<12	2.56 (1.09–6.25)	2.70 (1.03–7.69)	2.13 (0.76–5.88)	2.38 (0.71–8.33)
Vascular invasion				
No	1	1	1	1
Yes	3.78 (1.12–12.91)	3.19 (0.72–14.15)	3.43 (0.87–13.58)	2.91 (0.59–14.31)
Lymphatic invasion				
No	1	1	1	1
Yes	1.71 (0.63–4.67)	0.83 (0.19–3.69)	0.88 (0.26–2.94)	0.85 (0.09–2.89)
Site				
Right or trasversum	1	1	1	1
Left or double	2.69 (1.04–6.93)	2.06 (0.70–6.04)	3.56 (1.24–10.21)	3.34 (0.98–11.84)
Chemotherapy				
No	1	1	1	1
Yes	1.12 (0.38–3.34)	0.76 (0.17–3.37)	1.52 (0.39–5.97)	1.37 0.24–7.99)

*HR* hazard ratio

*CI* confidential interval

## Discussion

The main potential benefit of implementing follow-up programs is the early detection and subsequent treatment of disease relapse particularly when recurrence is asymptomatic [20]. The major challenge for any health care provider is to find a reasonable balance between optimal time intervals of surveillance visits and radiological imaging and health care resources used to provide these examinations. If on one side, patients derive a sense of well-being and reassurance with regular follow-up, on the other a strict surveillance may create anxiety prior to their visit and potential of false positive tests may result in further concern.

Recently several scientific societies and groups of experts provided guidelines and recommendations for the optimal strategies to implement follow-up of colon cancer [16, 21, 22]. One of the potential limitations is the lack of stratification of recurrent risk among different cancer stages, since the above recommendations are generically stated for all cancer stages. It is well recognized that early stages of colon cancer have a lower risk of relapse, and therefore, more accurate strategies for optimizing and tailoring surveillance protocols might be proposed. A risk-adapted follow-up in which the intensity varies according to the risk of recurrence might increase the cost-effectiveness by concentrating resources on patients at high risk. Selective use of intensive follow-up regimens, excluding patients at low risk for recurrence and patients who cannot tolerate further curative resection while concentrating on subpopulations of patients at high risk, may increase the utility of such regimens and thus improve efficacy. Continued improvements in risk stratification, disease detection, and treatment might increase the benefits of postoperative surveillance.

In this line of thought, we analyzed the overall and disease-free survival rates of patients with stage I, IIA, and IIB to evaluate if among these subjects we might find specific variables useful to identify cohorts with low or high risk of recurrence in order to split subgroups in which follow-up may be more of less intensive in term of time intervals of visits and diagnostic workup.

Our 5-year follow-up data are in accordance with recent large series [23] reporting overall and disease-free survival rates. Recurrent time and site were also quite consistent with other reports [21, 24]. In our series, 82 % of the patients were diagnosed with recurrence within 2 years and in all but one case, relapse was detected by US or CT scan.

By substage analyses, we found that overall and disease-free survival curves of stage I and IIA were almost overlapping while stage IIB had a negative impact on cancer-related mortality. With the intrinsic limitation of the small number of patients in our series, the present results confirm that stage IIB should be considered as a subgroup with poorer prognosis [25].

By analyzing the impact of single risk factors on recurrence, we observed that left or double site cancer were negative prognostic variables. This relationship between relapse and tumor location is not clear, but it may be related to tumor biology. One specific aspect of tumor biology is microsatellite instability (MSI). Multiple studies [5, 26] found that patients with MSI-positive tumors have a better overall prognosis and that MSI status is an independent positive predictor of survival. MSI is predominantly seen in right-sided colon cancers, and less than 5 % of left-sided cancers show MSI. Probably, in the next future, it will be necessary to include routinely MSI description in pathologic specimens to obtain a higher prognostic performance.

Several investigators aimed to identify a specific “ideal minimum” number of nodes to be reported. There has been great variation between studies in terms of identifying an “ideal minimum” number of nodes to be examined in stage II cancers, with figures ranging from 6 to 17 nodes [27–29]. An accurate description of node involvement may be particularly important in stage II tumors for a number of reasons. Firstly, the identification of a specific figure provides an indicator as to where it can be stated with defined certainty that the risk of long-term mortality is reduced [28, 29]. Secondly, it provides an important means of institutional quality control. Interestingly, the American National Quality Forum has endorsed a number of 12 nodes as a standard for assessing hospital performance in the surgical management of colonic tumors [27, 30]. Finally, an ideal minimum number of nodes may also be used as a “cut-off” value, below which stage II patients may be offered adjuvant chemotherapy [27].

In the present reports, the rate of patients with less than 12 nodes retrieved was 22 %. This figure is in line with other large series [31, 32], advocating that this is a common and worldwide problem, since this represents a negative prognostic factor [3, 33] as also suggested by our data.

Recently, tumor budding has been reported as one of the major malignant characteristics of colorectal carcinomas. It is defined as the presence of isolated single cells or small cell clusters (up to four cells) scattered in the stroma at invasive fronts; this represents feature loss of both glandular differentiation and cell cohesion that is crucial for the development of high invasive properties [6, 34]. Ueno classification divided it into high grade budding (≥10 foci) or low grade (<10 foci).

Lai et al. [35] reported that, in his experience, tumor budding is an important prognostic parameter for cancer-specific outcomes in patients with stage II colorectal cancer. They concluded that tumor budding could help to identify high-risk patients who might benefit from adjuvant chemotherapy. Othsuki et al. [36] demonstrated a significant difference in disease-free survival using our definition (high grade ≥10 foci, low grade <10 foci). Likely, our negative results were related to the relative low number of patients or for the inclusion of patient in stage I, and the exclusion of rectal cancer.

The presence and the degree of fibrosis in the cancer seems to be another important prognostic negative factor [37]. Mesker et al. [10] proposed a differentiation into high percentage (≥50 % of fibrosis) and low percentage (<50 %). Huijbers et al. [9] in a series of 710 patients observed a significant decrease in the overall survival and disease-free survival for the patients with high percentage of fibrosis versus patients with a low percentage of fibrosis (69 vs. 83 % and 58 vs. 77 %, respectively) but without differentiating the prognostic ability by stage. Our results suggest that, at least, in early colon cancer stages, fibrosis is not a key prognostic element.

In 2010, the European Society of Medical Oncology stated that the use of adjuvant chemotherapy in stage II CC remains controversial. In general, stage IIA can be considered low risk while stage IIB deserves adjuvant treatment. In stage IIA adjuvant treatment may be considered in the presence of unfavorable prognostic factors: obstruction or perforation debut, contiguity infiltration of neighboring organs, high grading, number of lymph nodes examined <12 and vascular, perineural or lymphatic infiltration [25]. Our policy was to candidate patients to adjuvant chemotherapy in stage IIB and in stage IIA only when at least two of the above risk factors were present. This conservative policy was based on the recommendations available at the time the study initiated. In fact, evidence from randomized controlled trials did not support the use of adjuvant chemotherapy, even for patients with high-risk stage II colon cancer [38] or at most when at least two risk factors were present [39].

## Conclusions

In conclusion, the present results suggest that staging remains of paramount importance in detecting patients at high risk of recurrence and in assigning a correct prognosis of long-term survival. The number of lymph nodes retrieved, the presence of vascular invasion, and the left or multiple cancer location are negative prognostic factors and should be take into account for the possibility of additional therapies. The detection of fibrosis and budding do not seem to add valuable information for prognosis. In early colon cancer stages, the surveillance program should be maximized within the first 2 years.
